# Depression and anxiety in transgender and non-binary adolescents: prevalence and associations between adolescent and caregiver reports

**DOI:** 10.1007/s00431-024-05723-z

**Published:** 2024-08-28

**Authors:** Sophia M. Liles, Anna L. Olsavsky, Diane Chen, Connor Grannis, Kristen R. Hoskinson, Scott F. Leibowitz, Eric E. Nelson, Charis J. Stanek, John F. Strang, Leena Nahata

**Affiliations:** 1 The Abigail Wexner Research Institute at Nationwide Children’s Hospital, 431 S 18th St,, Columbus, OH 43205, USA; 2 The Ohio State University College of Medicine, Columbus, OH, USA; 3 Ann and Robert H. Lurie Children’s Hospital, Northwestern University Feinberg School of Medicine, Chicago, IL, USA; 4 Nationwide Children’s Hospital, Columbus, OH, USA; 5 Center for Neuroscience, Children’s National Research Institute, Children’s National Hospital, Washington, DC, USA; 6 Department of Pediatrics, Neurology, and Psychiatry, George Washington University School of Medicine, Washington, DC, USA

**Keywords:** Mental health symptoms, Caregiver awareness, Transgender

## Abstract

Transgender/non-binary (TNB) adolescents are at increased risk for mental health concerns, and caregiver awareness is important to facilitate access to care. Yet, limited research has examined caregiver awareness of TNB mental health. Thus, we examined (1) the prevalence of internalizing symptoms (depression, generalized anxiety, separation anxiety, social anxiety) among TNB adolescents and (2) associations between adolescent and caregiver reports of adolescent mental health symptoms. TNB adolescents (*N* = 75) aged 12–18 and a caregiver were recruited from a multidisciplinary gender clinic in Ohio. Adolescents self-reported their mental health symptoms via the CDI and SCARED. Caregivers reported their perceptions of the adolescent’s mental health symptoms via the CASI-5. Descriptive statistics assessed participant characteristics, adolescent self-reported mental health symptoms, and caregiver proxy reports of adolescent mental health symptoms. Pearson’s correlations and scatterplots were used to compare adolescent and caregiver reports and McNemar tests assessed if the differences were statistically significant. Most TNB adolescents reported elevated symptoms of depression (59%), generalized anxiety (75%), separation anxiety (52%), and social anxiety (78%). Caregiver and adolescent reports were significantly correlated for depression (*r* = .36, *p* = .002), separation anxiety (*r* = .39, *p* < .001), and social anxiety (*r* = .47, *p* < .001). Caregiver and adolescent reports of generalized anxiety were not significantly correlated (*r* = .21, *p* = .08). McNemar tests were significant (all *p* < .001), such that adolescents’ reports met clinical cutoffs far more than their caregivers’ reports. *Conclusions*: Though adolescent and caregiver reports were low to moderately correlated, youth reports were consistently higher, suggesting the importance of interventions to increase caregiver understanding of TNB adolescent mental health.

## Introduction

The number of documented transgender and non-binary (TNB) adolescents in the US has increased in recent years; an estimated 1.4% of people ages 13–17 identify as transgender or non-binary [1], with variability seen based on study type [2]. TNB adolescents have significant and unique mental health concerns. With regard to internalizing mental health diagnoses, up to 50–62% of TNB adolescents experience depression and up to 27–68% experience generalized anxiety compared to 4–10% and 3–9% of adolescents in the general population who experience depression and generalized anxiety, respectively [3–7]. Other types of anxiety, such as separation and social anxiety, have not been well-studied in TNB populations, and thus, the prevalence rates of these conditions are unknown. Given the high rates of mental health concerns in TNB adolescents [3], it is important to better understand how specific mental health conditions impact this population to better inform treatments. It is important to note that TNB identities themselves should not be considered a causal factor for poor mental health; rather, as the gender minority stress model [8] proposes, stigma and lack of social support may be driving factors underlying elevated mental health challenges in TNB youth [9].

In pediatric populations, caregiver perspectives of their child’s mental health are important, as clinicians often rely heavily, or even solely, on caregiver reports to better understand the psychological functioning of adolescents in various contexts [10, 11]. Evidence suggests the most comprehensive and accurate clinical assessments integrate reports from multiple informants and TNB-specific standards of care recommend the involvement of caregivers in the assessment and treatment of TNB adolescents [12, 13]. However, research in general pediatric populations shows caregivers and adolescents often provide discrepant descriptions of the adolescents’ mental health symptoms, with only small to moderate correlations [10]. Though results have been mixed [11, 14–18], most studies show that when caregivers provide proxy reports for adolescents, caregivers report *more* internalizing symptoms than adolescents [11, 16–18].

Past research with general populations has demonstrated that discrepancies between caregivers and adolescents’ reports of adolescent mental health symptoms are associated with worse mental health treatment outcomes, with caregivers often reporting more symptoms than adolescents [19]. Given that TNB adolescents are at high risk for mental health concerns [3, 4, 6], it is important to understand potential caregiver–child discrepancies on TNB adolescents’ mental health, in particular the degree to which caregivers identify mental health symptoms. In the limited research published on caregiver–TNB adolescent dyads, TNB adolescents have self-reported more depression and general anxiety symptoms than proxy-reports by their caregivers [20, 21]. These data suggest caregivers of TNB adolescents may not fully recognize their child’s mental health needs. This is particularly problematic given the (a) high rates of psychological distress in this population [3]; (b) evidence showing supportive caregivers can act as an important buffer against negative mental health outcomes for TNB adolescents [22, 23]; and (c) reliance on caregivers to access care and consent to gender-affirming treatments [24], which have been associated with improved mental health outcomes of TNB adolescents [25, 26]. In this context, we examined (1) the prevalence of elevated internalizing symptoms, measured separately as depression, generalized anxiety, separation anxiety, and social anxiety among TNB adolescents and (2) associations between adolescent and caregiver reports of these adolescent mental health symptoms. We hypothesized that the rates of mental health concerns would be high and that there would be discrepancies between adolescent and caregiver reports.

## Methods

### Participants

Participants were recruited as part of a larger cross-sectional study from a gender development clinic (which provides multidisciplinary care to families of TNB adolescents) at a US pediatric hospital in Ohio. The study was approved by the Nationwide Children’s Hospital IRB (IRB18-00741) and conducted in accordance with the Declaration of Helsinki. Data were collected from December 2018–March 2020 and March 2021–Feburary 2022, with a lapse in recruitment due to COVID-19 restrictions on clinical research. For the larger study, eligible participants included adolescents ages 9–21 who had a diagnosis of gender dysphoria and were able to undergo an MRI for a separate component of the larger study. Participants were eligible regardless of treatment stage (e.g., puberty blockers, hormones, or no gender affirming medical interventions). Participants were required to have a caregiver informant available and willing to participate in the study. Participants provided written informed consent (if 18 years or older) or assent (if younger than 18) prior to data collection, with caregivers consenting for minors (those under 18 years of age). Of 101 participants approached, 82 agreed to participate (81% recruitment rate). For these analyses, only adolescents between 12 and 18 years of age were included due to the age range of the mental health measure validation: four caregiver–adolescent dyads were excluded due to missing survey data on either adolescent or caregiver mental health reports, leaving a final sample of 75 caregiver–adolescent dyads.

### Measures

Adolescents self-reported their mental health symptoms of depression, generalized anxiety, separation anxiety, and social anxiety. Adolescents completed the Children’s Depression Inventory (CDI) [27] to report their depressive symptoms. The tool is a validated measure for ages 7–17, though past research has also used the tool for 18 year olds [28]. The CDI is 27 items, and for each item, respondents are instructed to pick one of three sentences that best describe how they have felt in the prior 2 weeks. Depending on the sentence the participant selects, they are given 0–2 points that are summed to create a total score of 0–54, with higher scores indicating more depression symptoms. For example, participants would be asked to select one of the following: “*I am sad once in a while*” (0 points), “*I am sad many times*” (1 point), or “*I am sad all the time*” (3 points). The CDI is used primarily as a screening tool, has a clinical cutoff of 15 for clinical populations [29], and has previously been used with transgender adolescents [30].

To assess the three anxiety disorders, adolescents completed the Screen for Child Anxiety Related Emotional Disorders (SCARED), a validated measure commonly used to assess adolescents for anxiety disorders [31] that has been previously used with TNB adolescents [32]. The current analyses used only the subscales for generalized anxiety (nine items), separation anxiety (eight items), and social anxiety (seven items), based on availability of parallel scales for caregivers in this study. Example items include *I am nervous* (generalized anxiety), *I follow my mother or father wherever they go* (separation anxiety), and *I don’t like to be with people I don’t know well* (social anxiety). Each item is scored from 0 (*not true or hardly true*) to 2 (*very true or often true*) with higher scores indicating more anxiety symptoms. The subscales have maximum sum scores of 18, 16, and 14 for generalized anxiety, separation anxiety, and social anxiety respectively. The validated clinical cutoffs for each subscale are sum scores as follows: ≥ 9 for generalized anxiety, ≥ 5 for separation anxiety, and ≥ 8 for social anxiety. Cronbach’s alphas for the SCARED subscales in this sample ranged from *α* = 0.80–0.88, demonstrating strong internal consistency.

Caregivers completed the Child and Adolescent Symptom Inventory-5 (CASI-5) to report on their child’s mental health symptoms based on the DSM-5 diagnostic criteria [33]. This validated measure is typically employed as a screening tool [34] and has previously been used with parents of TNB adolescents [35]. The CASI-5 has 142 questions, divided into 37 total subscales, though these analyses used the major depressive episode (seven items), generalized anxiety (six items), separation anxiety (eight items), and social anxiety subscales (four items). Example items include *is depressed for most of the day* (depression), *has difficulty controlling worries* (generalized anxiety), *afraid to go to sleep unless near parent* (separation anxiety), and *is excessively shy with peers* (social anxiety). Participants rate each item from 0 (*never*) to 3 (*very often*), with maximum sum scores of 21, 18, 24, and 12 for depression, generalized anxiety, separation anxiety, and social anxiety, respectively. Higher scores indicate more mental health symptoms. The following scales and validated clinical cutoffs were used in this study: ≥ 6 for major depressive episode, ≥ 4 for generalized anxiety, ≥ 3 for separation anxiety, and ≥ 2 for social anxiety [33]. This sample had strong internal reliability on the CASI-5, with alpha levels ranging from *α* = 0.88–0.92.

### Analytic plan

All analyses were conducted in SPSS version 28. Descriptive statistics were used to assess participant characteristics, adolescent self-reported mental health symptoms, and caregiver proxy reports of adolescent mental health symptoms. We compared caregiver and self-report symptoms in each mental health category (depression, generalized anxiety, separation anxiety, and social anxiety) in two ways, though we were limited in the analyses possible because adolescents and caregivers completed different measures. However, previous works on caregiver–adolescent informant discrepancies have utilized distinct measures for youth and caregivers [36, 37]. First, Pearson’s correlations and scatterplots were used to directly compare relative values obtained from self and caregiver symptom reports. Second, since all symptom measures had objective and validated clinical cutoff scores, we binarized reports into “clinically significant” and “non-clinically significant” for each diagnostic category. We then used McNemar tests to assess if observed differences between self and caregiver determination of symptoms as “clinically significant” were statistically significant. We assessed significance as *p* < 0.05 and used listwise deletion for missing data, though data from no more than *n* = 2 (3%) of the sample was missing for any analysis.

## Results

All participant characteristics can be found in Table 1. Adolescent participants were between 12 and 18 years of age (*M* = 15.75, *SD* = 1.64). Adolescents were assigned female at birth (AFAB; *n* = 47; 63%) or assigned male at birth (AMAB; *n* = 28; 37%) and identified as male (*n* = 45; 60%), female (*n* = 23; 31%), or non-binary (*n* = 7; 9%). Of the seven non-binary participants, six were AMAB (86%), and one was AFAB (14%). Over half (*n* = 41, 55%) of the adolescent participants were receiving gender-affirming hormonal interventions at the time of the study (Table 1); 8 (11%) were treated with puberty blockers only, 30 (40%) were treated with hormone therapy only, 2 (3%) were treated with puberty suppression and hormones, and 35 (47%) were not treated with puberty blockers or hormones.

### Aim 1

**Fig. 1:**
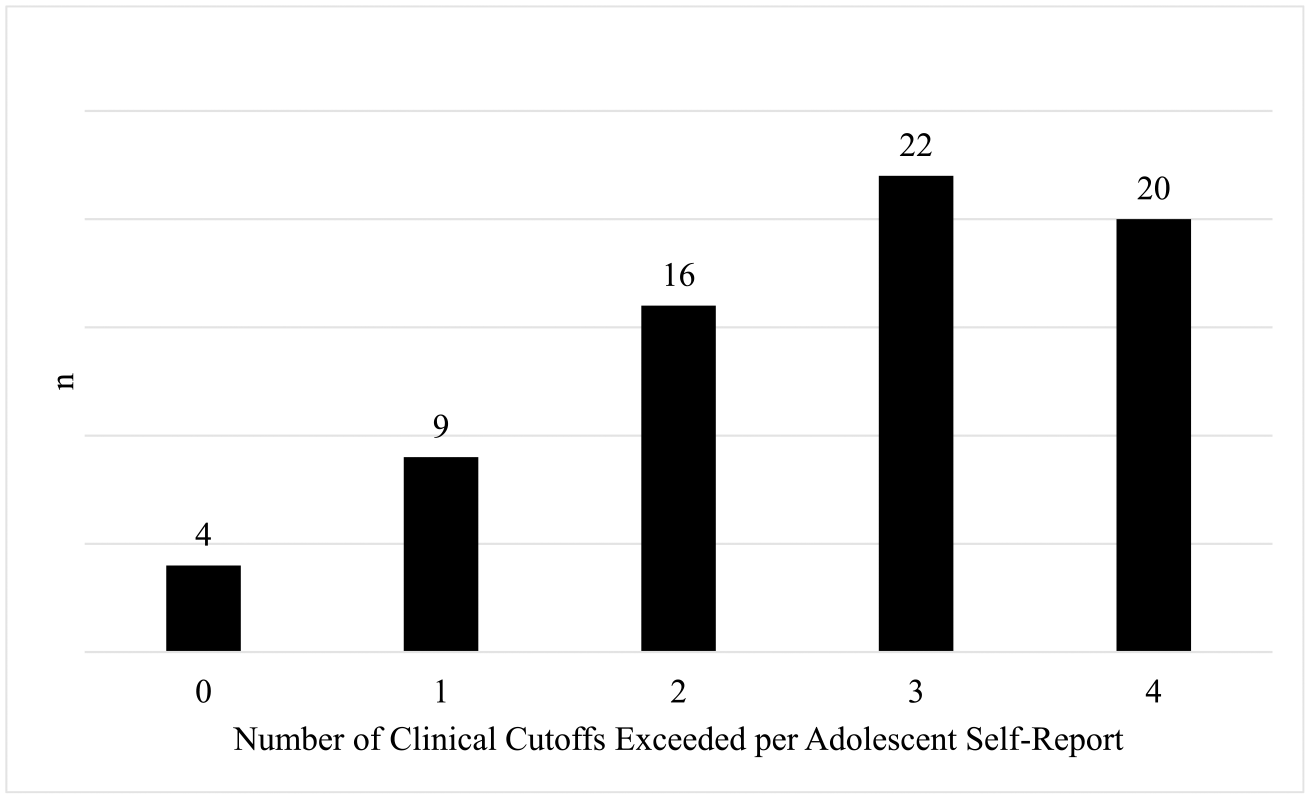
Number of adolescents who self-reported meeting multiple clinical cutoffs

Adolescents reported high levels of depression, generalized anxiety, separation anxiety, and social anxiety (Table 2). For all four mental health conditions, the majority of adolescents reported symptoms exceeding the clinical cutoffs; 59% met the depression cutoff, 75% met the generalized anxiety cutoff, 52% met the separation anxiety cutoff, and 78% met the social anxiety cutoff. Of the 73 adolescents who had complete mental health data, 95% (*n* = 69) reported symptoms that exceeded the clinical cutoff for at least one mental health concern and 27% (*n* = 20) reported symptoms that exceeded the clinical cutoff for all four mental health concerns (Fig. 1). Caregiver reports of adolescent mental health can be found in Table 2.

### Aim 2

**Fig. 2:**
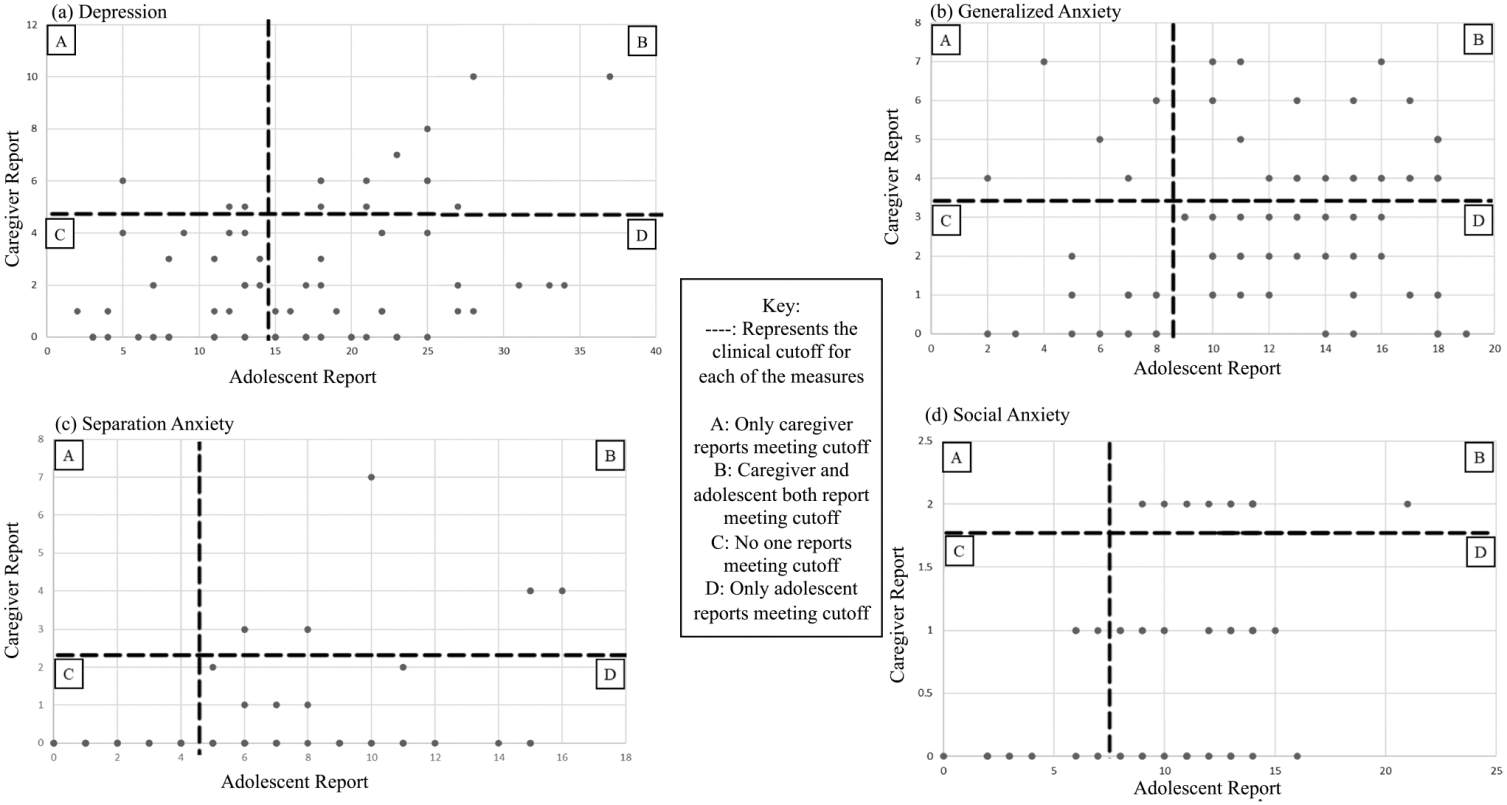
Scatterplots of adolescent self-report and caregiver proxy report of adolescent mental health

Caregiver and adolescent reports were significantly correlated for depression (*r* = 0.36, *p* = 0.002), separation anxiety (*r* = 0.39, *p* < 0.001), and social anxiety (*r* = 0.47, *p* < 0.001). Caregiver and adolescent reports of generalized anxiety were not significantly correlated (*r* = 0.21, *p* = 0.08). Scatterplots depicting adolescent and caregiver reports of adolescent mental health can be found in Fig. 2. McNemar tests were significant (all *p* < 0.001), such that adolescents’ reports met clinical cutoffs far more than their caregivers’ report (Table 3; Fig. 2).

## Discussion

This study expands the range of diagnostic mental health categories examined in TNB adolescents to include specific subtypes of anxiety and provides parallel information about caregiver awareness of TNB adolescent mental health. As hypothesized, the majority of adolescents in our sample exceeded clinical cutoffs for depression, generalized anxiety, separation anxiety, and social anxiety. Despite finding small-moderate correlations between adolescent and caregiver reports across most categories, adolescents reported far *more* internalizing symptoms than their caregivers, which is a distinct pattern compared to most prior research conducted in cisgender populations [11, 16–18, 38].

Self-reported symptom levels met established clinical cutoffs for clinical diagnoses for most adolescents in this sample, corroborating findings from some previous research that TNB adolescents have substantially worse mental health than their cisgender peers [3, 7]. Over half of adolescents reported depressive symptoms above the clinical cutoff, which is similar to past findings [3, 4]. Additionally, three quarters of TNB adolescents in this study reported clinically concerning symptoms of generalized anxiety, which is more than previously reported (27–68%) [3, 6, 7] and could in part reflect local sociopolitical factors such as anti-TNB legislation [9] and effects of the COVID-19 pandemic [39]. Furthermore, this study contributes novel information regarding specific anxiety disorders, as separation anxiety and social anxiety have not been well-studied in the TNB population [6]. Though separation anxiety has not been thoroughly investigated in adolescents, research does show elevated rates of separation anxiety in TNB children, and it is hypothesized that these elevated rates are related to overall high rates of internalizing problems [40]. There have been calls to expand the range of diagnoses investigated to aid in the development of targeted psychological interventions for TNB youth [41], and further research is needed to identify factors contributing to the high levels of separation and social anxiety found in this study.

One possible reason for increased internalizing symptoms in TNB populations stems from factors described in the gender minority stress model [8], which emphasizes stigma and perceived lack of social support TNB teens face in their everyday lives. Both the stigma and perception of social ostracism has been made worse by recent anti-TNB legislation [9]; thus, the risk level for mental health concerns in this population may continue to increase. Recent developments which restrict access to gender-affirming care, often including mental health care, across the US increases this risk [42]. Additionally, gender dysphoria [43] and family dynamics [44] could be contributing to the increased mental health concerns in TNB adolescent populations.

Adolescent and caregiver-proxy reports were generally low to moderately correlated, with adolescents consistently reporting far more symptoms than their caregivers; the strongest correlation was seen in social anxiety reports (perhaps due to observable behavioral changes) while generalized anxiety reports were not significantly correlated. These findings suggest that caregivers may not be fully aware of their child’s mental health status, particularly regarding internalizing symptoms that are less apparent. Though the strength of correlations is similar to prior literature, these findings differ from research with cisgender populations that have mostly found adolescents report fewer symptoms than their caregivers [11, 17]. The current findings are similar to past research that has found TNB adolescents report more depression and generalized anxiety symptoms than their caregivers [20, 21] and expand the current literature by including caregiver awareness for separation and social anxiety. Caregiver awareness has been associated with family relationship quality, family functioning, child insight, caregiver engagement, and caregiver–adolescent communication in presumed cisgender populations [14, 45], and research suggests TNB adolescents may be less likely than cisgender adolescents to have strong family relationships [46]. Additionally, it is important to note that many caregivers of TNB adolescents carry a high mental load due to isolation, stigma, anxiety about their child’s future wellbeing, and the constant need to advocate for their child [38, 47] that could interfere with fully recognizing their child’s mental health status. Thus, these family factors may explain why TNB adolescents report far more mental health symptoms than their caregivers recognize, but further research is needed to confirm these associations.

The study findings must be interpreted in the context of several limitations. Adolescents in this sample were brought to a gender-affirming clinic by a caregiver and were likely to have access to resources not available to other TNB adolescents in the community (family support, access to a clinic, financial resources). Adolescent mental health and caregiver awareness of adolescent mental health may be poorer among those without access to a gender clinic or without the emotional support of family [48]. It is also possible that TNB adolescents in a multidisciplinary gender clinic have higher levels of distress than TNB adolescents who are not seeking care, as they may have higher levels of distress that prompted seeking treatment at the gender clinic [49]. Additionally, some adolescents in a gender clinic may conceal mental health symptoms, as they may believe mental health symptoms could lead some caregivers to withhold consent for treatment [21]. Further research with a community sample of TNB adolescents is needed to better understand the mental health of adolescents with varying levels of social support and at different stages of transitioning. Additionally, the majority of adolescents were transmasculine, the majority of caregivers were mothers, and all participants were primarily white, limiting the generalizability of the findings to other populations. Adolescents and caregivers also completed different mental health measures, substantially limiting the statistical tests that could be examined. For example, because discrepancies between adolescent and caregiver reports were analyzed descriptively, it was not possible to assess associations between gender-affirming treatment status and discrepancy, as doing so would have required rescaling the adolescent and caregiver measures. Lastly, the cross-sectional nature limits the conclusions that can be made; because adolescent mental health was not measured before and after the onset of gender dysphoria and/or treatment initiation, it is not possible to know the role of gender dysphoria onset or treatment in the mental health of TNB adolescents and/or discrepancy with caregivers. Despite these limitations, this study suggests that caregivers of TNB adolescents are often not fully aware of their child’s mental health, the consequences of which are particularly worrisome due to their high-risk status.

In summary, the high prevalence of mental health symptoms in TNB adolescents and lack of caregiver awareness of these symptoms has both clinical and research implications. TNB adolescents should receive routine mental health screening with particular attention to depression and anxiety (generalized anxiety, social anxiety, separation anxiety). Although many clinicians obtain both caregiver and adolescent self-reports [10, 11], our findings suggest that adolescent self-reports should be prioritized alongside caregiver reports as stated in a recent research review recommendation [50], so that if either party reports elevated mental health symptoms, the adolescent will receive timely referrals for mental health support. Caregiver psychoeducation on mental health risks and signs of mental health concerns may help improve caregiver awareness of adolescent mental health, and holistic family-centered care should be provided. Increased caregiver awareness of mental health symptoms has implications for improved family relationships, caregiver–child communication, and access to mental health treatment and gender-affirming care for minors [14, 19, 22, 45, 48]. Longitudinal studies with a diverse group of caregiver–TNB adolescent dyads are needed to better understand the relationship between caregiver awareness and adolescent mental health over time, with the ultimate goal of developing family-centered interventions to improve health outcomes in TNB adolescents.

## Figures and Tables

**Table 1:** Demographic and treatment characteristics

	*N*	%
Adolescent age (years)	Mean = 15.75	*SD* = 1.64
Adolescent gender identity		
Male	45	60.00
Female	23	30.67
Non-binary	7	9.33
Adolescent assigned gender at birth		
Male	28	37.33
Female	47	62.67
Adolescent race		
White	54	72.00
Bi/multiracial	11	14.67
Black or African American	3	4.00
Native American or American Indian	1	1.33
Prefer not to answer/skipped question	6	8.0
Adolescent ethnicity		
Hispanic or Latino	5	6.67
Not Hispanic or Latino	70	93.33
Caregiver relationship to child		
Mothers	49	65.33
Fathers	16	21.33
Other primary caregivers	5	6.67
Did not disclose relationship to child	5	6.67
Gender-affirming hormonal interventions		
Hormones	30	40.00
Puberty blocker	8	10.67
Both hormones and puberty blockers	2	2.67
No medical intervention	35	46.67

**Table 2:** Adolescent and caregiver mental health reports

		*M*(*SD*)	Clinical cutoff	*n* (%) who met clinical cutoff
Depression	Adolescent report	16.49 (8.11)	15/54	44 (58.67%)
	Caregiver report	2.23 (2.56)	6/21	8 (10.96%)
Generalized anxiety	Adolescent report	12.00 (4.53)	9/18	55 (75.34%)
	Caregiver report	2.79 (2.09)	4/18	18 (24.00%)
Separation anxiety	Adolescent report	5.66 (4.02)	5/16	38 (52.06%)
	Caregiver report	0.40 (1.18)	3/24	5 (6.85%)
Social anxiety	Adolescent report	10.18 (4.13)	8/14	57 (78.08%)
	Caregiver report	0.67 (0.78)	2/12	14 (19.18%)

**Table 3:** McNemar tests of adolescent and caregiver reports of meeting clinical cutoffs

		Caregiver report does NOT meet clinical cutoff (*n*)	Caregiver report meets clinical cutoff (*n*)
Depression*	Adolescent report does NOT meet clinical cutoff (*n*)	29	1
	Adolescent report meets the clinical cutoff (*n*)	36	7
Generalized anxiety*	Adolescent report does NOT meet clinical cutoff (*n*)	15	3
	Adolescent report meets the clinical cutoff (*n*)	38	15
Separation anxiety*	Adolescent report does NOT meet clinical cutoff (*n*)	33	0
	Adolescent report meets the clinical cutoff (*n*)	33	5
Social anxiety*	Adolescent report does NOT meet clinical cutoff (*n*)	16	0
	Adolescent report meets the clinical cutoff (*n*)	41	14

* *p* < 0.001

## Data Availability

Data may be available on request from the authors, with consideration of privacy/ethical restrictions.

## What is Known:

- Transgender/non-binary adolescents are at high risk for mental health concerns and caregivers are essential to coordinate care.

## What is New:

- This study expands the diagnostic mental health sub-categories examined in transgender/non-binary adolescents, noting elevated symptoms of separation and social anxiety.
- Transgender/non-binary adolescents reported more symptoms of depression, generalized anxiety, separation anxiety, and social anxiety than caregivers.

## Abbreviations

AFAB: Assigned female at birth
AGAB: Assigned gender at birth
AMAB: Assigned male at birth
CASI-5: Child and Adolescent Symptom Inventory-5
CDI: Child Depression Inventory
TNB: Transgender and non-binary
